# Railway mortality for several mammal species increases with train speed, proximity to water, and track curvature

**DOI:** 10.1038/s41598-020-77321-6

**Published:** 2020-11-24

**Authors:** Colleen Cassady St. Clair, Jesse Whittington, Anne Forshner, Aditya Gangadharan, David N. Laskin

**Affiliations:** 1 Department of Biological Sciences, University of Alberta, Edmonton, T6G 2E9 Canada; 2 Parks Canada Agency, Banff National Park, Banff, AB T1L 1K2 Canada; 3 Parks Canada Agency, Banff, Kootenay, and Yoho National Parks, Lake Louise, AB T0L 1E0 Canada

**Keywords:** Conservation biology, Conservation biology

## Abstract

Railways are a major source of direct mortality for many populations of large mammals, but they have been less studied or mitigated than roads. We evaluated temporal and spatial factors affecting mortality risk using 646 railway mortality incidents for 11 mammal species collected over 24 years throughout Banff and Yoho National Parks, Canada. We divided species into three guilds (bears, other carnivores, and ungulates), compared site attributes of topography, land cover, and train operation between mortality and paired random locations at four spatial scales, and described temporal patterns or mortality. Mortality risk increased across multiple guilds and spatial scales with maximum train speed and higher track curvature, both suggesting problems with train detection, and in areas with high proximity to and amount of water, both suggesting limitations to animal movement. Mortality risk was also correlated, but more varied among guilds and spatial scales, with shrub cover, topographic complexity, and proximity to sidings and roads. Seasonally, mortality rates were highest in winter for ungulates and other carnivores, and in late spring for bears, respectively. Our results suggest that effective mitigation could address train speed or detectability by wildlife, especially at sites with high track curvature that are near water or attractive habitat.

## Introduction

Direct mortality from collisions with vehicles is a global and increasing problem on both roads^1,2^ and railways^3,4^, that can be substantial enough to reduce the viability of threatened populations such as Asian elephants (*Elephas maximus*)^5^, grizzly bears (*Ursus arctos*)^6^, and Florida panthers (*Puma concolor coryi*)^7^. Consequently, mitigation of transportation corridors that pass through wildlife habitat is an important priority for species conservation around the world^8^. An early step in mitigation planning for wildlife is to determine whether mitigation should be site- or time-specific, vs. continuous and permanent. Site-specific mitigation can protect critical habitat, increase connectivity, and reduce mortality risk^9–11^. Alternatively, time-specific mitigations, such as nighttime road closures, can benefit wildlife^12^, particularly during seasons of greater activity^13^. These options are generally quicker and less expensive to implement than spatially continuous and permanent mitigation, which is often achieved via exclusion fencing that is perforated with crossing structures^14,15^. It is important to support mitigation decisions with robust logic and evidence because of their significant commitments of time, money and political capital^16,17^.

Several factors potentially influence whether transportation mitigation should be generic and extensive or specific and more intensive, either spatially or temporally. Extensive mitigation has emerged as the ideal form in affluent jurisdictions trying to prevent fragmentation of populations and widespread wildlife mortality, particularly in protected areas with charismatic, threatened, wide-ranging populations^16^. Extensive mitigation is also more prevalent where animal-vehicle collisions impose a direct cost to humans via damage to vehicles and injuries to people^18^. Mitigation that is spatially or temporally specific is more likely to occur when extensive mitigations are too costly ^1^ or where one or a few charismatic, endangered species are vulnerable to vehicle collisions in predictable locations. Examples of this specificity include amphibian migrations^19^, turtle nesting movements near roads^20^, and migratory corridors for ungulates^21^. The ecological value of species-specific mitigations can be significantly increased, still with relatively low economic costs, if they serve multiple species^10^, which can sometimes be achieved with umbrella species with large home ranges^10,22^.

Unfortunately, several ecological factors could limit the generality of mortality mitigation among species for planning mitigation locations, even within a taxonomic grouping, such as large mammals for which sensitivity to habitat modification can be surprisingly diverse^23^. On the other hand, predatory or scavenging behaviour may increase the spatial overlap (and hence mortality risk from vehicles) between carnivores and herbivores^24^, as many landscape features such as drainages channel the movement of multiple species^14^. Investigating these processes to determine congruence in mortality hotspots of multiple taxa could support the most cost-effective and ecologically beneficial mitigation while identifying the strengths, as well as limitations, of management actions dedicated to focal species^10^.

Decisions about implementation of extensive and generic vs. intensive and specific mitigations for a transportation corridor depend on many factors including whether mitigations are to be applied to roads or railways. For roads, the extensive approach has already emerged as the clear gold standard for jurisdictions that can afford it^25^, but wildlife fencing may impose negative effects in areas with lower vehicle density^26^, particularly on roads with few crossing structures^27^. Railway mitigation has hardly begun^28,29^, but some authors advocate the same extensive approach for mitigating railways^3^. Two factors limit the viability of expensive fencing and crossing structures in this context. First, mortality can increase sharply where fences end and animals can access the transportation corridor^30,31^. Fence ends are frequently mitigated on roads with grates that deter animal movement, but without complete efficacy^32^, and railways require surface continuity. Second, collisions with wildlife on railways are less likely to injure people, or be witnessed by them, reducing societal demand for expensive mitigation. These factors increase the logic of site-specific mitigations in areas of heightened mortality rates or risk to populations of conservation or cultural concern. Such hotspots of collisions have been studied extensively on roads^33^, but similar comparative study for railways is still relatively rare^34,35^. There is not yet a general understanding of the spatial and temporal factors that increase mortality risk for wildlife on railways and how those factors vary among taxa.

The objectives of this paper were to (a) identify the landscape factors and seasons that increase train-caused mortality for large mammals and (b) determine the similarity of these explanatory variables among guilds as a means to assess and prioritize the broader conservation value of potential mitigations. Our study was motivated partly by public attention to train-caused mortality of grizzly bears in two Canadian mountain parks where correlates of bear attraction and mortality have been studied extensively^36^. We used 24-years of wildlife-train collision data to evaluate spatial and temporal factors affecting collision risk for eleven species of large mammals that we grouped into three guilds. We expected that mortality risk would increase in (a) places where animals had difficulty perceiving trains, (b) where their movement paths were constrained by adjacent topography or water, (c) where forage opportunities increased, or (d) where the railway provided security from encounters with people; we developed predictor variables to test these hypotheses. Our goal was to support implementation of railway mitigations, in this and similar mountainous areas, corresponding to the times and locations of greatest risk.

## Materials and methods

### Study area

**Figure 1:**
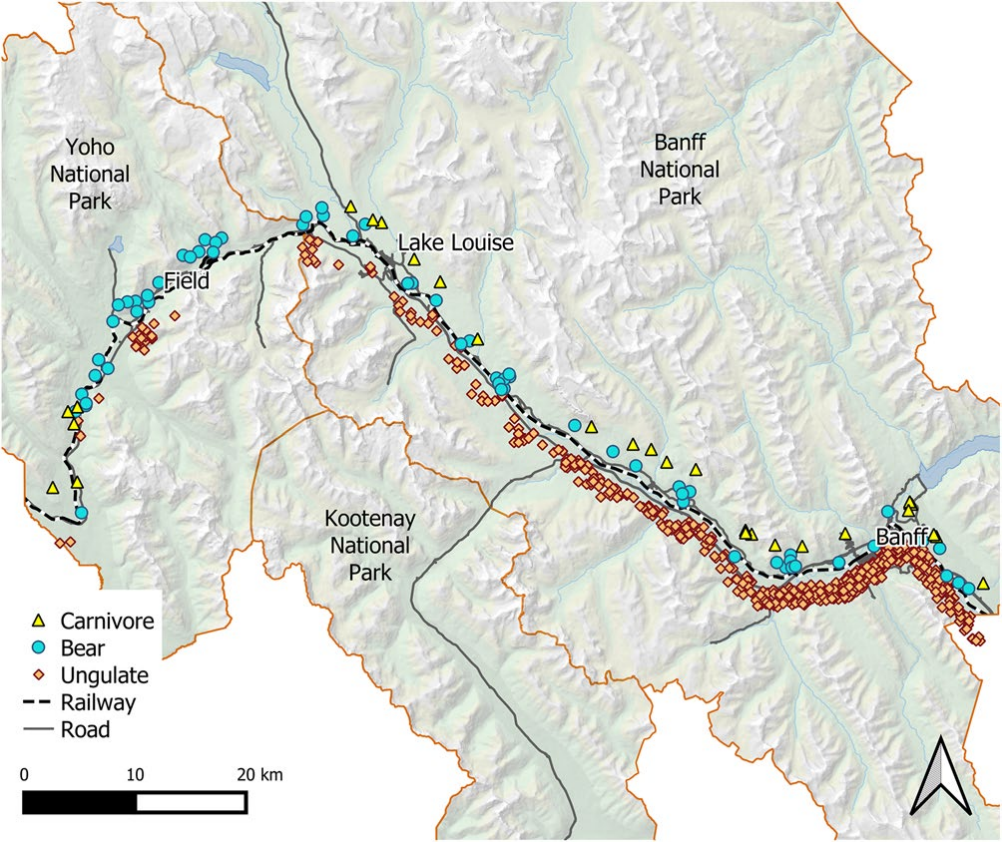
Location of Banff and Yoho National Parks, Canada, the railway track and roads that pass through them, and the sites of mortalities (offset from the rail) for three guilds of mammals recorded with handheld GPS between 1995 and 2018. The map was created by the authors with QGIS (version 3.10.0 https://www.qgis.org).

The study was conducted in Banff and Yoho National Parks, which are located in the Rocky Mountains of Alberta, Canada (Fig. 1). Our study occurred within a busy transportation corridor that contains the Canadian Pacific Railway (134 km), the adjacent TransCanada Highway, three towns, major rivers, steep topography, a utility corridor, several secondary roads and dozens of human-use areas. The TransCanada highway, which receives over eight million vehicles a year^37^, is fenced through the entire length of Banff National Park. Construction of 44 wildlife crossing structures located on average every two km have reduced wildlife mortality rates and increased connectivity^38^. Similar mitigations are underway in Yoho National Park.

The railway has been operational since 1881 and is used to ship agricultural commodities such as grain^39^ and a variety of other products (potash, consumer goods, vehicles, forestry products, and refined fuel products) between the interior of Canada and ports on the western coast. Approximately 20 trains were measured to pass through the study area daily^40^, but the number is typically a little higher during the fall shipping season (J. Van Humbeck, Canadian Pacific Railway, personal communication). Information about variation in train traffic between day and night was not available. Human use adjacent to the railway included towns, ski hills, golf courses, hiking trails, campgrounds, day-use areas, and areas of current or past operational use, such as garbage dumps and gravel pits. Elevational declines occur in both directions from the continental divide that separates Banff and Yoho National Parks and these lower elevation areas contain higher densities of many species, especially ungulates in the east end of Banff^4^.

### Field methods

We used a long-term database (1995–2018) from Parks Canada Agency (hereafter PCA), consisting of 646 confirmed wildlife mortalities. We used only those records that were initially reported by train crews under an obligatory reporting system and later substantiated via site visits by PCA personnel who (a) confirmed the presence of a carcass, (b) determined its species, sex and age class, and (c) spatially identified its location with a handheld GPS (Fig. 1). We used confirmed mortality events as the unit of replication in our analysis even if more than one animal was killed during the event. For each event, we recorded the species, number of animals, date and time of mortality as well as spatial coordinates. Some records included only a single time stamp, which we assumed to correspond with the collision, but potentially corresponding to the reporting, thereby limiting the interpretation of these data. Data were groomed to identify GPS errors and snapped to the railway if they fell within 100 m of the railway. We included data from the following species for analysis: grizzly bears, black bears (*Ursus americanus*), wolves (*Canis lupus*), coyotes (*Canis latrans*), cougar (*Puma concolor*), lynx (*Lynx canadensis*), moose (*Alces alces*), elk (*Cervus canadensis*), white-tailed deer (*Odocoileus virginianus*), mule deer (*O. hemionus*), unspecified deer, and bighorn sheep (*Ovis canadensis*) (Table 1).

**Table 1:** Number of confirmed mortality events with GPS coordinates for each species on the railway through Banff and Yoho National Parks from 1995 through 2018.

Guild	Species	Mortality	Mortality by guild
Bears	Black bear	47	59
	Grizzly	12	
Carnivores	Wolf	15	27
	Coyote	8	
	Cougar	3	
	Lynx	1	
Ungulates	Elk	328	560
	Whitetail deer	116	
	Mule deer	66	
	Moose	29	
	Deer spp.	15	
	Sheep	6	

### Analytical methods

To identify the explanatory variables that best explained locations where mortality events occurred, we compared mortality locations to *available* locations at four spatial scales for each of three guilds; *bears*, *other carnivores*, and *ungulates*. First, we built logistic regression models with 5000 random locations distributed along the railway throughout the study area (an average of one random location every 26 m). This model assessed where animals were killed within our study area. The distribution of mortality locations could be influenced by variability in animal density over space and time. For example, the population of elk declined by 75% in about 2000 and shifted their distribution eastward in winter toward the Banff townsite^4^. We therefore developed additional models that compared the habitat attributes of mortality locations to paired locations on the railway within a one day’s travel. We constrained available points to occur within each of 2.5 km, 5 km, and 10 km of a mortality location to accommodate habitat use at multiple spatial scales^41^. We derived these scales from the daily mean (range = 3.4 km for black bears to 13.8 km for wolves) and upper 95% quantiles (range = 7.5 for black bears to 23.7 km for wolves) of movement distances from GPS-collared animal in our study area (after^42^). We used conditional logistic regression to compare mortality locations to paired random locations on the rail with a 1:100 ratio of cases to controls.

We used four types of explanatory landscape variables to model the spatial characteristics of mortality events and grouped these according to animal perception of trains, channelling of animal movement, forage quality, and security from people (Table 2). Several variables pertained to more than one potential causative relationship and we offer the following hypotheses as guides to the logic of model predictions that were not mutually exclusive. We hypothesized that the ability of an animal to perceive an approaching train would reduce strike risk such that mortality rates would increase with increasing track curvature, change in elevation, posted train speed, and canopy cover. We also hypothesized that features that channel animal movement onto the railway would increase risk of collisions, predicting mortality rates would increase with topographic complexity, the amount or proximity of water, proximity of roads, and canopy closure. We hypothesized that high forage quality in the vicinity of the railway would increase animal attraction, and hence the risk of strikes, predicting positive correlations with proximity to railway sidings (where trains travel slowly such that leaking grain accumulates^39^) and with higher shrub cover, which provides berry-producing forage for bears and browse for ungulates. Finally, we hypothesized that strikes would increase where animals spent more time because they had high security from people in the busy valley bottoms of these protected areas, predicting that mortality would increase with distance to roads, shrub density, and canopy cover. We calculated each landscape variable at three spatial scales (with radii of 90, 210 and 390 m based on 30 m resolution base layers). We did not use larger radii for these analyses to maximize our ability to discriminate between cases and their controls (above). For each variable we selected the radius that produced the best fit to the data with all species combined to improve comparisons across guilds and analytical scales. We removed variables that were highly correlated (r > 0.6) and that had high variance inflation factors.

**Table 2:** Explanatory variables used in modelling animal-train collisions for three guilds of large mammals (bears, other carnivores, and ungulates) in Banff and Yoho National Parks, Canada between 1995 and 2018, along with the hypothesized mechanism by which these variables may influence probability of collision.

Covariate	Hypothesized mechanism	Description
Rail curvature	*Detection* obscured of approaching trains	Curvature (tortuosity) = line length (L) divided by net displacement (R) (for L equal to 1000 m). Spiral tunnels omitted
Topographic complexity	*Detection* and *movement* channeled by topography or barriers	Terrain rugosity, or the density of changes in slope across space (Ardron 2002). Source: 20 m DEM. Scale = 90 m radius
Distance to water	*Movement*	Distance in km to nearest edge of water feature (i.e. lake, river). Source: Landsat 7-based landcover map (McDermid 2007) Parks Canada hydrology layers
Water cover	*Movement*	Percent water cover within a 90 m radius. Source: remote sensing based landcover map + Parks Canada hydrology layers
Distance to roads	*Movement* and *security* from humans	Distance to roads in km, sidings a proxy for increased availability of spilled grain (attractant)^39^
Distance to sidings	*Forage* availability increases	Distance to rail sidings in km, sidings a proxy for increased availability of spilled grain (attractant)^39^
Shrub cover	*Forage* and *security*	Percent shrub cover within a 390 m radius. Source: Landsat 7-based based landcover map
Canopy closure	*Detection, movement, security, forage*	Average percent canopy closure within a 210 m radius. Source: Landsat 7-based based landcover map
Winter/summer	*Movement* and *forage*	Indicator variable that equals 1 for winter and − 1 for summer (sine)
Spring/autumn	*Forage*	Indicator variable that equals 1 for spring and − 1 for autumn (cosine)
Snow on ground	*Movement*	Daily measurement of total accumulated snow at Banff CS meteorological station (cm) Source: Environment Canada
Recent precipitation	*Movement*	Daily measurement of 24 h snowfall at Banff CS meteorological station (cm) Source: Environment Canada
MaxSpeed	*Detection*	Maximum allowable train speed (km) posted by railway

For each guild and spatial scale of available points, we constructed a full model with all landscape variables as main effects. We included four biologically plausible, two-way interactions between track curvature and each of maximum train speed, percent water cover, distance to water, and percent shrub cover. We ran models with all combinations of variables while requiring a minimum of ten mortality events per covariate to reduce the likelihood of overfitting^43^. We ranked models using Bayesian information criterion (BIC) and selected models within two BIC values of the best model. We avoided perils of averaging model coefficients^44^ and instead visually presented model coefficients for all parameters in the top models with parameters ordered by model weights across guilds and scales^45^. We provided parameter estimates from the top ranked model. We assessed model fit using the area under the receiver operating characteristic curve (ROC) for the top logistic regression model for the whole study area.

We assessed the effects of season, precipitation, and time of day on mortality risk. For each day from 1995 to 2018, we determined whether an animal from each guild died (no = 0, yes = 1). We then used logistic regression to assess the effects of season (sine and cosine of year day), total precipitation (mm), and the interaction between total precipitation and winter (snow) on mortality risk. Day of year is a circular variable so we used sine of year day (spring = 1, fall = − 1) and cosine of year day (winter = 1, summer = − 1). We again compared models using BIC. We expected that that mortality risk would increase in seasons when deep snows, high water levels, and new precipitation made the railway more attractive for travel (winter) and when spilled grain and early emergent rail-side vegetation had higher nutritional quality and availability compared to broader food availability (winter and early spring) (daily precipitation; Table 2). We lacked spatial predictions for precipitation and snow accumulation data for the duration of our study, so we used metrics estimated at the Banff Meteorological Station. Precipitation varied throughout our study area with areas near the continental divide having had more precipitation and cooler temperatures. We tallied the timing of mortality events by hour via the reporting that was provided in the PCA database, and divided them into approximate periods of day (0800 h–1959 h) and night (2000 h–0759 h). We offer limited interpretation because records were sometimes missing this information and these times may sometimes represent the timing of reporting, rather than the timing of collisions.

All analyses were conducted in R 3.6.1^46^ and the package survival 3.1.8 ^47^.

## Results

**Figure 2:**
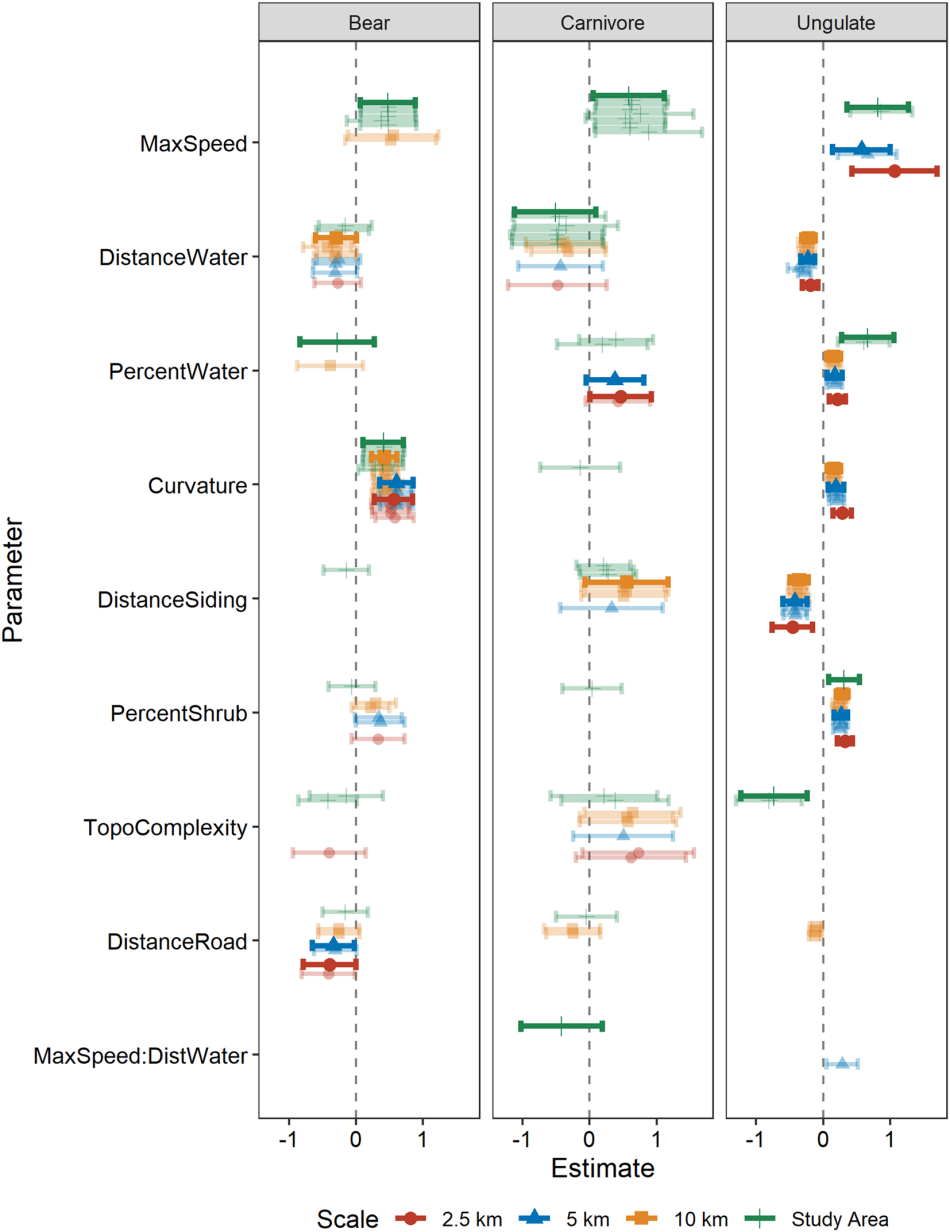
Parameter estimates and 95% CI’s for all models with ΔBIC ≤ 2 (light colours) at four spatial scales (km) describing mortality locations on a railway for three guilds of large mammals, bears, other carnivores, and ungulates, in Banff and Yoho National Parks, Canada. Parameters are ordered by frequency of occurrence across guilds from top to bottom. Dark colours identify parameter estimates for the top model for each guild and scale.

Our data set included 59 bear mortalities, 27 other carnivores, and 560 ungulates for a total of 646 events for 11 species of large mammals (Table 1). We used conventional and conditional logistic regression to evaluate the effect of predictor variables we expected to be associated with one or more of (a) the ability of animals to perceive trains, (b) a channelling effect on animal movement, (c) forage opportunities via vegetation or prey that are attracted to it, or (d) security from people (Table 2). The number of candidate models within 2 BIC of the top model in the four scales of analysis ranged from one to five for ungulates, five to nine for bears, and four to nine for carnivores (Table 3), perhaps owing to the smaller sample sizes and the limited number of covariates allowed per model. The direction of parameter effects was consistent among spatial scales, but it sometimes differed among guilds (Fig. 2).

**Table 3:** Model selection for factors affecting wildlife railroad mortality assessed using random locations distributed throughout the study area using logistic regression and within scale specific distances of paired mortality sites using conditional logistic regression.

Guild	Scale	df	BIC	ΔBIC	Weight	Model
Bear	Study area	4	220.4	0.0	0.17	MaxSpeed + Curvature + PercentWater
		4	220.5	0.2	0.15	MaxSpeed + Curvature + DistanceRoad
		4	220.6	0.3	0.15	MaxSpeed + Curvature + DistanceSiding
		4	220.7	0.4	0.14	MaxSpeed + DistanceWater + Curvature
		4	221.1	0.7	0.11	MaxSpeed + Curvature + TopoComplexity
		4	221.2	0.9	0.11	MaxSpeed + Curvature + Canopy
		4	221.3	0.9	0.11	MaxSpeed + Curvature + PercentShrub
		4	222.2	1.8	0.07	DistanceWater + Curvature + TopoComplexity
	10 km	2	533.5	0.0	0.15	Curvature + Canopy
		2	533.8	0.3	0.13	Curvature + DistanceWater
		1	533.9	0.4	0.12	Curvature
		3	534.4	0.9	0.10	Curvature + DistanceWater + Canopy
		3	534.5	1.0	0.09	Curvature + DistanceWater + PercentShrub
		2	535.0	1.5	0.07	Curvature + DistanceRoad
		3	535.0	1.5	0.07	Curvature + DistanceWater + PercentWater
		3	535.0	1.5	0.07	Curvature + DistanceWater + DistanceRoad
		3	535.2	1.7	0.07	Curvature + DistanceWater + MaxSpeed
		4	535.4	1.9	0.06	Curvature + DistanceWater + Canopy + PercentWater
		3	535.4	2.0	0.06	Curvature + Canopy + MaxSpeed
	5 km	2	527.3	0.0	0.34	Curvature + Canopy
		2	527.9	0.6	0.25	Curvature + DistanceRoad
		1	528.9	1.6	0.15	Curvature
		3	529.2	1.9	0.13	Curvature + Canopy + DistanceRoad
		3	529.3	2.0	0.13	Curvature + DistanceWater + DistanceRoad
	2.5 km	2	535.2	0.0	0.29	Curvature + Canopy
		2	535.4	0.3	0.25	Curvature + DistanceRoad
		1	535.6	0.4	0.24	Curvature
		2	537.0	1.8	0.11	Curvature + PercentShrub
		2	537.1	2.0	0.11	Curvature + DistanceWater
Carnivore	Study Area	4	141.1	0.0	0.12	MaxSpeed + DistanceWater + MaxSpeed:DistWater
		4	141.1	0.0	0.12	MaxSpeed + DistanceWater + Canopy
		4	141.9	0.7	0.08	MaxSpeed + DistanceWater + DistanceSiding
		4	142.0	0.9	0.08	MaxSpeed + PercentWater + DistanceSiding
		4	142.0	0.9	0.08	MaxSpeed + Canopy + PercentWater
		4	142.2	1.1	0.07	MaxSpeed + Canopy + DistanceSiding
		4	142.3	1.2	0.07	MaxSpeed + Canopy + PercentShrub
		4	142.4	1.3	0.06	MaxSpeed + Canopy + MaxSpeed:Canopy
		4	142.5	1.4	0.06	MaxSpeed + DistanceWater + PercentWater
		4	142.5	1.4	0.06	MaxSpeed + DistanceWater + TopoComplexity
		4	142.6	1.5	0.06	MaxSpeed + DistanceWater + Curvature
		4	142.8	1.7	0.05	MaxSpeed + DistanceWater + DistanceRoad
		4	142.8	1.7	0.05	MaxSpeed + DistanceWater + PercentShrub
		4	143.0	1.9	0.05	MaxSpeed + TopoComplexity + DistanceSiding
	10 km	1	249.4	0.0	0.21	DistanceSiding
		1	249.6	0.2	0.19	TopoComplexity
		1	250.3	1.0	0.13	DistanceWater
		2	250.3	1.0	0.13	DistanceSiding + TopoComplexity
		1	250.9	1.5	0.10	DistanceRoad
		2	251.1	1.7	0.09	DistanceRoad + DistanceSiding
		2	251.1	1.7	0.09	DistanceWater + DistanceSiding
		2	251.3	1.9	0.08	DistanceWater + TopoComplexity
	5 km	1	250.0	0.0	0.34	PercentWater
		1	250.2	0.2	0.30	DistanceWater
		1	250.8	0.8	0.22	TopoComplexity
		1	251.8	1.8	0.14	DistanceSiding
	2.5 km	1	249.1	0.0	0.35	PercentWater
		1	249.6	0.4	0.28	TopoComplexity
		2	250.2	1.1	0.20	PercentWater + TopoComplexity
		1	250.6	1.4	0.17	DistanceWater
Ungulate	Study Area	5	490.1	0.0	0.42	MaxSpeed + PercentWater + TopoComplexity + PercentShrub
		5	490.3	0.1	0.39	MaxSpeed + Canopy + PercentWater + TopoComplexity
		4	491.6	1.5	0.20	MaxSpeed + PercentWater + TopoComplexity
	10 km	8	5078.8	0.0	0.39	Curvature + DistanceWater + Canopy + PercentWater + DistanceSiding + Curvature:Canopy + PercentShrub + Curvature:PercentShrub
		9	5079.8	0.9	0.25	Curvature + DistanceWater + Canopy + DistanceRoad + PercentWater + DistanceSiding + Curvature:Canopy + PercentShrub + Curvature:PercentShrub
		7	5080.2	1.4	0.20	Curvature + DistanceWater + Canopy + DistanceRoad + PercentWater + DistanceSiding + Curvature:Canopy
		8	5080.7	1.9	0.16	Curvature + DistanceWater + Canopy + DistanceRoad + DistanceSiding + Curvature:Canopy + PercentShrub + Curvature:PercentShrub
	5 km	8	5106.9	0.0	0.50	Curvature + DistanceWater + Canopy + PercentWater + DistanceSiding + Curvature:Canopy + PercentShrub + Curvature:PercentShrub
		9	5106.9	0.0	0.50	Curvature + DistanceWater + Canopy + PercentWater + DistanceSiding + Curvature:Canopy + PercentShrub + Curvature:PercentShrub + MaxSpeed
	2.5 km	6	5099.6	0.0	0.67	Curvature + Canopy + DistanceRoad + PercentWater + Curvature:Canopy + MaxSpeed
		5	5101.0	1.4	0.33	Curvature + Canopy + DistanceRoad + PercentWater + Curvature:Canopy

Top ranked models were ≤ 2 ΔBIC of the top model.

When we ranked parameters by their frequency within the top models and consistency in positive or negative responses, the best predictor of mortality sites was maximum train speed, followed by proximity to water, amount of water within 90 m, and track curvature (Tables 3 and 4, Fig. 2). Mortality risk increased with maximum posted train speed in top models at the scale of the study area for all three guilds and for ungulates at finer scales. Mortality risk increased near water for all guilds but was not always in the top bear and carnivore models. The percent of water increased mortality risk for carnivores and ungulates, but marginally reduced risk for bears (Fig. 2). Curvature increased mortality risk at all spatial scales for bears and ungulates, but not other carnivores.

**Table 4:** Parameter estimates for factors affecting wildlife railroad mortality comparing mortality and available locations using logistic regression (study area) and conditional logistic regression (paired random and mortality locations).

Guild	Scale	Parameter	Estimate	SE	Statistic	P-value
Bear	Study Area	Curvature	0.408	0.154	2.643	0.008
	Study Area	MaxSpeed	0.477	0.209	2.284	0.022
	Study Area	PercentWater	− 0.278	0.283	− 0.981	0.327
	Study Area	Intercept	− 0.673	0.181	− 3.714	0.000
	10 km	Curvature	0.423	0.095	4.433	0.000
	10 km	DistanceWater	− 0.294	0.156	− 1.888	0.059
	5 km	Curvature	0.608	0.127	4.775	0.000
	5 km	DistanceRoad	− 0.337	0.160	− 2.100	0.036
	2.5 km	Curvature	0.561	0.146	3.854	0.000
	2.5 km	DistanceRoad	− 0.394	0.202	− 1.953	0.051
Carnivore	Study Area	MaxSpeed	0.588	0.271	2.172	0.030
	Study Area	MaxSpeed:DistWater	− 0.419	0.310	− 1.353	0.176
	Study Area	DistanceWater	− 0.510	0.309	− 1.646	0.100
	Study Area	Intercept	− 1.514	0.254	− 5.971	0.000
	10 km	DistanceSiding	0.554	0.317	1.748	0.081
	5 km	PercentWater	0.377	0.220	1.714	0.086
	2.5 km	PercentWater	0.465	0.236	1.970	0.049
Ungulate	Study Area	Intercept	1.076	0.140	7.699	0.000
	Study Area	MaxSpeed	0.815	0.232	3.511	0.000
	Study Area	PercentWater	0.665	0.200	3.323	0.001
	Study Area	PercentShrub	0.312	0.116	2.696	0.007
	Study Area	TopoComplexity	− 0.735	0.253	− 2.909	0.004
	10 km	PercentShrub	0.286	0.046	6.272	0.000
	10 km	Curvature	0.161	0.052	3.095	0.002
	10 km	PercentWater	0.156	0.055	2.821	0.005
	10 km	DistanceWater	− 0.217	0.052	− 4.159	0.000
	10 km	DistanceSiding	− 0.359	0.070	− 5.140	0.000
	5 km	PercentShrub	0.266	0.052	5.141	0.000
	5 km	Curvature	0.191	0.058	3.315	0.001
	5 km	PercentWater	0.171	0.057	2.983	0.003
	5 km	MaxSpeed	0.570	0.220	2.589	0.010
	5 km	DistanceWater	− 0.232	0.053	− 4.336	0.000
	5 km	DistanceSiding	− 0.420	0.092	− 4.574	0.000
	2.5 km	PercentShrub	0.328	0.057	5.720	0.000
	2.5 km	Curvature	0.282	0.070	4.024	0.000
	2.5 km	PercentWater	0.212	0.063	3.373	0.001
	2.5 km	MaxSpeed	1.063	0.325	3.272	0.001
	2.5 km	DistanceSiding	− 0.457	0.154	− 2.961	0.003
	2.5 km	DistanceWater	− 0.193	0.060	− 3.220	0.001

The results show the top ranked model for each guild and spatial scale.

Five lower-ranked variables in the top models exhibited less consistency among guilds and spatial scales (Tables 3 and 4, Fig. 2). Ungulates exhibited higher risk of mortality nearer to sidings, whereas risk for other carnivores increased with distance to sidings. Mortality increased with percent of shrub cover at all spatial scales for ungulates and bears. At the spatial scale of the study area, ungulates and bears had higher mortality where topographical complexity was lower, but topographical complexity increased mortality risk for carnivores at all scales. All guilds exhibited a tendency for greater mortality near roads, but this parameter occurred across spatial scales only for bears. An interaction suggested that the combined effects of train speed and proximity to water was most pronounced for other carnivores (Fig. 2). The logistic regression models with random points distributed along the railway throughout the study area did a moderate to poor job at differentiating mortality sites from random locations. Area under the ROC curve (AUC) was slightly higher for ungulates (AUC = 0.735) compared to bears (AUC = 0.634) and other carnivores (AUC = 0.683).

**Figure 3:**
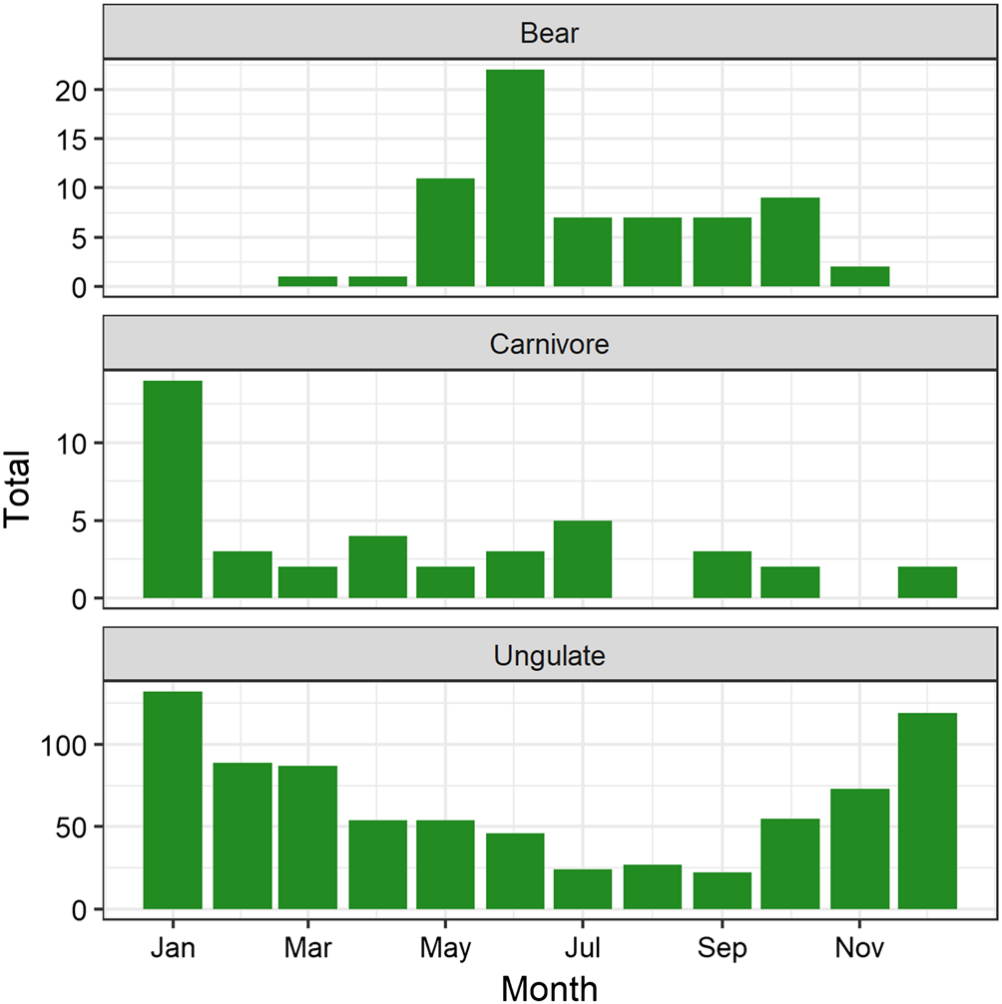
Total cumulative mortalities by month between 1995 and 2018 for three guilds of large mammals struck on the railway through Banff and Yoho National Parks, Canada.

**Figure 4:**
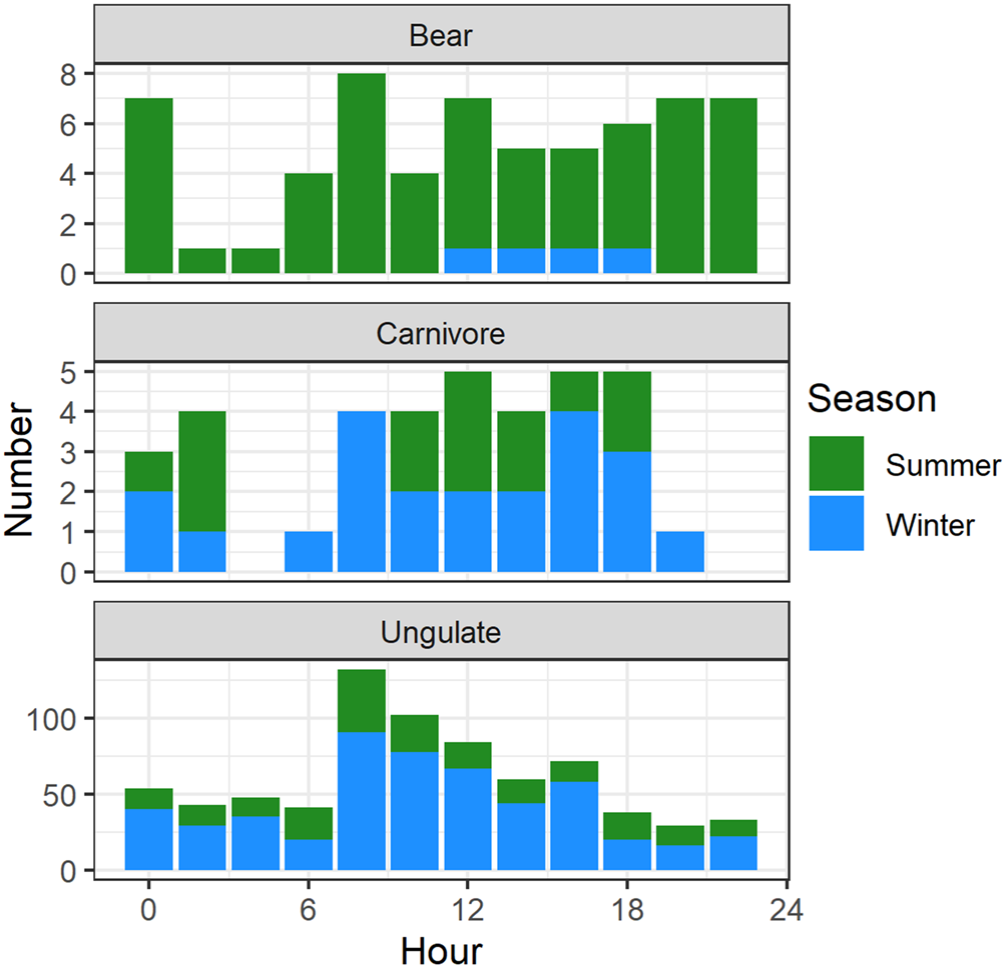
Number of mortalities recorded by hour of the day between 1995 and 2018 for three guilds of large mammals struck on the railway through Banff and Yoho National Parks, Canada.

Temporal patterns of mortality differed among species (Figs. 3 and 4). Seasonally, ungulate mortality increased in winter (YearDay_cosine_ = 0.583, SE = 0.063, z-value = 9.3) and spring (YearDay_sine_ = 0.309, SE = 0.063, z-value = 4.9). The second ranked model had a ΔBIC = 8.5. The top model for bears had no covariates, but the second ranked model with ΔBIC = 2.8 suggested bear mortality increased in summer (YearDay_cosine_ = − 0.751, SE = 0.330, z-value = − 2.3). Carnivore mortality did not change statistically throughout the year. The second ranked model had ΔBIC = 5.5 and a weak positive covariate for an increase in spring mortality. Precipitation and the precipitation-winter interaction were not important covariates for any species. Our tally of diel information revealed that fewer mortalities occurred (or were reported) as occurring at night than during the day: 43% of 54 events for bears, 28% of 25 events for other carnivores, and 37% of 537 events for ungulates occurred at night (Fig. 4).

## Discussion

Mitigating wildlife-train collisions is likely to be more affordable, both ecologically and economically, if it can be achieved with efforts that are limited spatially or temporally to the locations and times of greatest risk to wildlife^16,36^, but little guidance is available for identifying these foci or evaluating their congruence among species. We sought to advance this information by evaluating predictor variables at several spatial scales across three guilds of large mammals, bears, other carnivores, and ungulates, killed on the railway in two mountain protected areas of Canada. We identified four parameters, train speed, proximity to water, amount of water, and track curvature, that were robust predictors of mortality across guilds and spatial scales and five more parameters with lesser effects. Seasons of mortality risk were significantly higher for ungulates in mid to late winter, but were less pronounced for bears (slightly higher in late spring and early summer), and other carnivores (no strong seasonal effects). Wildlife mortality occurred more often during the day than night.

Two of the top-ranked parameters in our analysis, train speed and track curvature, likely affected mortality rates via failed detection of approaching trains. The positive effect of posted train speed on mortality was robust across all three guilds, but only at the largest (study area) spatial scale. The absence of this effect at smaller spatial scales may reflect the lesser variation in train speed over shorter distances. Several other studies of rail mortality also identified a positive effect of train speed^35,48,49^ and vehicle speed is broadly associated with wildlife-vehicle collisions on roads^50^. A plausible explanation for the ubiquity of this relationship is that fast vehicles simply overwhelm the sensory capacity, and hence motor response, of animals^51^. Track curvature was positively related to mortality at all spatial scales of analysis for bears and at the three smaller spatial scales of analysis for ungulates. Again, this effect is consistent with other studies of wildlife mortality on both roads^50,52^ and railways^35,53^. Another set of researchers working in our study area hypothesized that curvature would increase collision risk primarily if it reduces the ability for wildlife to detect approaching trains via acoustic cues^54^. Others have suggested that curvature could, instead, reduce risk if it causes slower vehicle speed^55^. Sensory-based limitations of detection may frequently contribute to wildlife-vehicle collisions, although they have received little direct study to date^51^.

Two other top-ranked correlates of mortality, proximity to and amount of water, are consistent with limitations to animal movement as trains approached. Proximity to water increased collision risk for all three guilds and at multiple spatial scales, but increasing amounts of water within 90 m increased mortality rates only for ungulates (all four spatial scales) and other carnivores (finer two spatial scales). Although bear mortality events had a weak, negative association with the percent of water, several strike sites for grizzly bears, which were less numerous than for black bears^4^, also occurred near water^56^. The rail may afford easier travel through difficult terrain^56,57^, including wet and boggy habitat. Water may interact with other variables, such as shrubby habitat, to influence risk via attraction to forage or habitat. In our study area, it is unlikely that animals were attracted to water as a limited resource as occurs for elephants in India^48^.

The other parameters that entered our top models had less consistent effects among guilds. Ungulate mortalities increased near railway sidings, perhaps because more grain accumulates where trains travel at slower speeds^39^. Elk congregated near the town of Banff, which contains a siding. However, mortalities of other carnivores occurred farther from sidings, perhaps because these wary animals avoid areas with people. The positive effect of shrub cover on bear and ungulate mortality is consistent with several mechanisms, including use of shrubs as a forage source or hiding cover, or correlation with wet habitat. Shrubs are favoured forage for many deer species^58^ and calving sites for elk^59^. Topographical complexity was negatively associated with ungulate mortality, probably because the species that predominated our dataset favour broad open valleys. Bear mortality increased with proximity to roads, maybe because of attraction to their verge habitat as forage^60,61^, or owing to smaller-scale avoidance of roads and associated human use at topographic pinch points^62^. Other carnivores exhibited higher mortality rates in association with higher train speeds when they were close to water, again indicative of an effect on escape behaviour.

The seasons of peak vulnerability for wildlife in our study area differed among guilds; bears were most vulnerable in late spring (June) whereas ungulates and other carnivores were more often struck in mid-winter. Winter peaks correspond with greater snow depth, which would make the railway an easier travel route for both groups, and lower forage availability, which could increase attraction by ungulates to train-spilled grain^39^. Elsewhere, winter is a season of greater collision frequency on railways for moose^63^, roe deer^34^, and elk^53^, similar to the patterns on roads for moose^64^. Bears may have exhibited peak mortality in late spring for several reasons; this is the season when 2 or 3-year-old bears are displaced by their mothers^65^, vegetation abundance and phenology is enhanced along the railway^66^, bears may use the railway to search for ungulate calves^36^, and water levels are at their highest in June, impeding movement near the railway. Bear mortality would be expected to peak in the fall if it is driven by grain spilled from rail cars^39^ and in early spring if it is driven by winter accumulation of grain or ungulate carcasses.

Our diel analysis showed that strikes are slightly more common during the day than at night, although it was not always clear in the database whether time stamps applied to the collision or reporting event. Moreover, our division into day and night periods was not adjusted by season. Railway collisions are more common at crepuscular periods for several European cervids^67^ and grey kangaroos (*Macropus giganteus*)^49^, while moose-train collisions increased at night and during full moons^63^. The capacity many animals have to increase nocturnality to avoid peaks in human activity^68^ may interact with adjacent human use to increase their vulnerability to night-travelling trains. More work on these temporal patterns is warranted and identifying the so-called hot moments for mortality^69^, could be as important to their mitigation as identifying their locations.

Our study had several limitations that affect its inferences. One was the highly unequal sample sizes among guilds, which limited the number of covariates allowed in the bear and other carnivore models, contributed to increased model uncertainty, and limited our statistical power to identify the most important variables affecting mortality risk. Obviously, more abundant species produce more robust statistical models, but these models may not always reflect risk factors for the rare species of greatest conservation concern. A second limitation is the use of mortality data alone to identify risk areas. Although analyses of collision hotspots^50^ and hot moments^69^ have dominated analyses for road mitigation, past mortality is not a good indicator of future mitigation if it caused local avoidance or reduced population sizes^70^. An alternate approach for identifying vulnerability might be to combine information from animal movement with information from collisions^71^. Application of this approach in our study area suggested that areas of high rail use by GPS-collared grizzly bears were negatively associated with past collision sites^56^. Future analyses might better integrate hazards of train movement with animal exposure^72^. A third limitation is that we did not measure all the variables that may predict mortality sites, such as the position of tributaries to the Bow River. Valley bottoms are predictive of landscape-level movement for many wide-ranging species and often predict mortality sites when incorporated in analyses^63,73^. A previous analysis of wildlife-train collisions in our study area found that proximity to movement barriers like snow sheds and bridges predicted collisions sites for bears^74^. We did not include measures of human use, which are known to affect the distribution of wary carnivores^13^. Our use of shrub cover as a variable precluded use of the correlated (but weaker) variable of forest cover, which has sometimes been positively associated with rail mortality^35,63^ and sometimes negatively^34^. Fourth, our guild-based unit of analysis overlooked species interactions, such as the avoidance of grizzly bears by black bears^75^. Finally, we may have underestimated the spatial scales of maximum relevance for our predictor variables (at 90, 210, and 390 m). However, recent work in our study area suggests these values were well within biologically relevant differences in escape time associated with provision of a warning system^40^. In that study, 55–110 m is the linear track distance that corresponded to the increased escape time measured for small animals (3.3 s) and large ones (6.5 s, respectively) with an average train speed of 60.5 km/h.

Despite these limitations, our results offer some insights for the planning of mitigation on this railway that might be generalized to other jurisdictions where similar precision of mortality data have not been collected. Key findings for mitigation that were robust across guilds and spatial scales related primarily to train detection and animal movement. Mortality increased with maximum train speed and track curvature, both assumed to reduce the ability of wildlife to detect approaching trains. Mortality also increased in areas with closer proximity to and more water that may impede animal movement, as well as increase access to forage or hiding cover. Interestingly, this combination of features characterizes two relative hotspots of grizzly bear mortality in our study area^56^. Further, seasons of vulnerability differed between bears and other wildlife, which has important implications for timing of mitigations.

Identifying the specific sites where mitigation is most needed could obviate the need for expansive mitigation consisting of fencing and crossing structures that is consistently recommended for high-traffic roads^1^. Although the same extensive approach could reduce wildlife mortality on railways, it is unlikely to be economically feasible because of the low likelihood of human injury. Further, fence intrusions in remote and inaccessible areas could substantially increase mortality risk. Our results support suggestions by others that reducing train speed could be a particularly effective mitigation and its economic cost could be reduced by concentrating it in areas where track curvature combines with impediments to animal movement. When such hotspots can be identified for species of conservation concern, they might be further mitigated by clearing attractive vegetation^76^, augmenting limiting resources in safer locations^48,76^, or installing warning systems^54,77,78^. Beyond more attention to the magnitude and correlates of wildlife mortality on railways, there is a tremendous need to better understand their indirect effects of habitat loss, fragmentation and barriers^79^, particularly for species that are less charismatic and less studied^80^.

## References

[1] Glista DJ, DeVault TL, DeWoody JA (2009). A review of mitigation measures for reducing wildlife mortality on roadways. Landsc. Urban Plan..

[2] Rytwinski, T. & Fahrig, L. The impacts of roads and traffic on terrestrial animal populations. in *Handbook of Road Ecology* (eds van der Ree, R., Smith, D. J. & Grilo, C.) 237–246. 10.1002/9781118568170 (Wiley Blackwell, 2015).

[3] Carvalho, F., Santos, S. M., Mira, A. & Lourenço, R. Methods to monitor and mitigate wildlife mortality in railways. in *Railway Ecology* (eds Borda-deAgua, L., Barriento, R., Beja, P., & Pereira, H. M.) 23–42. 10.1007/978-3-319-57496-7 (Springer, 2017).

[4] Gilhooly P, Nielsen SE, Whittington J, St Clair CC (2019). Wildlife mortality on roads and railways following highway mitigation. Ecosphere.

[5] Johnsingh AJT, Williams AC (1999). Elephant corridors in India: Lessons for other elephant range countries. Oryx.

[6] Waller JS, Servheen C (2005). Effects of transportation infrastructure on grizzly bears in northwestern Montana. J. Wildl. Manag..

[7] Schwab AC, Zandbergen PA (2011). Vehicle-related mortality and road crossing behavior of the Florida panther. Appl. Geogr..

[8] van der Ree, R., Smith, D. J. & Grilo, C. The ecological efects of linear infrastructure and traffic: challenges and opportunities of rapid global growth. in *Handbook of Road Ecology* (eds R. van der Ree, Smith D. J., & Grilo C.) 1–9. 10.1002/9781118568170 (Wiley Blackwell, 2015).

[9] Gunson, K. & Teixeira, F. Z. Road-wildlife mitigation planning can be improved by identifying the patterns and processes associated with wildlife-vehicle collisions. in *Handbook of Road Ecology* (eds van der Ree, R., Smith, D. J. & Grilo, C.) 101–109. 10.1002/9781118568170 (Wiley Blackwell, 2015).

[10] Polak T, Nicholson E, Grilo C, Bennett JR, Possingham HP (2019). Optimal planning to mitigate the impacts of roads on multiple species. J. Appl. Ecol..

[11] Sawaya MA, Kalinowski ST, Clevenger AP (2014). Genetic connectivity for two bear species at wildlife crossing structures in Banff National Park. Proc. R. Soc. B Biol. Sci..

[12] Gubbi S, Poornesha HC, Madhusudan MD (2012). Impact of vehicular traffic on the use of highway edges by large mammals in a South Indian wildlife reserve. Curr. Sci..

[13] Whittington J, Low P, Hunt B (2019). Temporal road closures improve habitat quality for wildlife. Sci. Rep..

[14] Clevenger AP, Chruszcz B, Gunson KE (2001). Highway mitigation fencing reduces wildlife-vehicle collisions. Wildl. Soc. Bull..

[15] Grilo, C., Smith, D. J. & Klar, N. Carnivores: Struggling for survival in roaded landscapes. in *Handbook of Road Ecology* (eds R. van der Ree, D. J. Smith, & C. Grilo) 300–312. 10.1002/9781118568170 (Wiley Blackwell, 2015).

[16] Huijser MP, Duffield JW, Clevenger AP, Ament RJ, McGowen PT (2009). Cost-benefit analyses of mitigation measures aimed at reducing collisions with large ungulates in the United States and Canada: A decision support tool. Ecol. Soc..

[17] Gurrutxaga M, Saura S (2014). Prioritizing highway defragmentation locations for restoring landscape connectivity. Environ. Conserv..

[18] Seiler A (2005). Predicting locations of moose-vehicle collisions in Sweden. J. Appl. Ecol..

[19] Woltz HW, Gibbs JP, Ducey PK (2008). Road crossing structures for amphibians and reptiles: Informing design through behavioral analysis. Biol. Conserv..

[20] Steen DA (2006). Relative vulnerability of female turtles to road mortality. Anim. Conserv..

[21] Seidler RG, Green DS, Beckmann JP (2018). Highways, crossing structures and risk: Behaviors of Greater Yellowstone pronghorn elucidate efficacy of road mitigation. Glob. Ecol. Conserv..

[22] Noss RF, Quigley HB, Hornocker MG, Merrill T, Paquet PC (1996). Conservation biology and carnivore conservation in the Rocky Mountains. Conserv. Biol..

[23] Gangadharan A, Vaidyanathan S, St Clair CC (2016). Categorizing species by niche characteristics can clarifyconservation planning in rapidly-developing landscapes. Anim. Conserv..

[24] Cook TC, Blumstein DT (2013). The omnivore's dilemma: Diet explains variation in vulnerability to vehicle collision mortality. Biol. Conserv..

[25] van der Grift EA (2013). Evaluating the effectiveness of road mitigation measures. Biodivers. Conserv..

[26] Jaeger JAG, Fahrig L (2004). Effects of road fencing on population persistence. Conserv. Biol..

[27] Huijser MP (2016). Effectiveness of short sections of wildlife fencing and crossing structures along highways in reducing wildlife-vehicle collisions and providing safe crossing opportunities for large mammals. Biol. Conserv..

[28] Popp JN, Boyle SP (2017). Railway ecology: Underrepresented in science?. Basic Appl. Ecol..

[29] Dorsey, B., Olsson, M. & Rew, L. J. Ecological effects of railways on wildlife. in *Handbook of Road Ecology* (eds R. VanderRee, D. J. Smith, & C. Grilo) 219–227. 10.1002/9781118568170 (Wiley Blackwell, 2015).

[30] Cserkesz T, Ottlecz B, Cserkesz-Nagy A, Farkas J (2013). Interchange as the main factor determining wildlife-vehicle collision hotspots on the fenced highways: spatial analysis and applications. Eur. J. Wildl. Res..

[31] Plante J, Jaeger JAG, Desrochers A (2019). How do landscape context and fences influence roadkill locations of small and medium-sized mammals?. J. Environ. Manag..

[32] Cramer, P. & Hamlin, R. *Testing New Technology to Restrict Wildlife Access to Highways: Phase 2*. (Utah. Dept. of Transportation, 2016).

[33] Teixeira FZ, Kindel A, Hartz SM, Mitchell S, Fahrig L (2017). When road-kill hotspots do not indicate the best sites for road-kill mitigation. J. Appl. Ecol..

[34] Kusta T (2014). Deer on the railway line: Spatiotemporal trends in mortality patterns of roe deer. Turk. J. Zool..

[35] Jasinska KD (2019). Linking habitat composition, local population densities and traffic characteristics to spatial patterns of ungulate-train collisions. J. Appl. Ecol..

[36] St Clair CC (2019). Animal learning may contribute to both problems and solutions for wildlife-train collisions. Philos. Trans. R. Soc. B Biol. Sci..

[37] Hunt, W. A. *Banff National Park State of the Park Report—Resource Conservation Technical Summaries 2008 to 2017* (2018).

[38] Clevenger AP, Chruszczc B, Gunson KE (2003). Spatial patterns and factors influencing small vertebrate fauna road-kill aggregations. Biol. Conserv..

[39] Gangadharan A (2017). Grain spilled from moving trains create a substantial wildlife attractant in protected areas. Anim. Conserv..

[40] Backs JAJ, Nychka JA, St Clair CC (2020). Warning systems triggered by trains increase flight-initiation times of wildlife. Transp. Res. Part D.

[41] DeCesare NJ (2012). Transcending scale dependence in identifying habitat with resource selection functions. Ecol. Appl..

[42] Rode KD (2014). Effects of capturing and collaring on polar bears: findings from long-term research on the southern Beaufort Sea population. Wildl. Res..

[43] Aho K, Derryberry D, Peterson T (2014). Model selection for ecologists: The worldviews of AIC and BIC. Ecology.

[44] Cade BS (2015). Model averaging and muddled multimodel inferences. Ecology.

[45] Dormann CF (2018). Model averaging in ecology: A review of Bayesian, information-theoretic, and tactical approaches for predictive inference. Ecol. Monogr..

[46] R Core Team. *R: A Language and Environment for Statistical Computing*, https://www.R-project.org/ (2019).

[47] Therneau, T. *A Package for Survival Analysis in S_version 2.38*. https://CRAN.R-project.org/package=survival (2015).

[48] Joshi R, Puri K (2019). Train-elephant collisions in a biodiversity-rich landscape: A case study from Rajaji National Park, north India. Hum.-Wildl. Interact..

[49] Visintin C, Golding N, van der Ree R, McCarthy MA (2018). Managing the timing and speed of vehicles reduces wildlife-transport collision risk. Transp. Res. Part D-Transp. Environ..

[50] Gunson KE, Mountrakis G, Quackenbush LJ (2011). Spatial wildlife-vehicle collision models: A review of current work and its application to transportation mitigation projects. J. Environ. Manag..

[51] Lima SL, Blackwell BF, DeVault TL, Fernández-Juricic E (2015). Animal reactions to oncoming vehicles: A conceptual review. Biol. Rev..

[52] Grilo C, Bissonette JA, Santos-Reis M (2009). Spatial-temporal patterns in Mediterranean carnivore road casualties: Consequences for mitigation. Biol. Conserv..

[53] Popp JN, Hamr J, Chan C, Mallory FF (2018). Elk (*Cervus elaphus*) railway mortality in Ontario. Can. J. Zool..

[54] Backs JAJ, Nychka JA, St Clair CC (2017). Warning systems triggered by trains could reduce collisions with wildlife. Ecol. Eng..

[55] Valero E, Picos J, Alvarez X (2015). Road and traffic factors correlated to wildlife-vehicle collisions in Galicia (Spain). Wildl. Res..

[56] Pollock SZ, Whittington J, Nielsen SE, Clair CCS (2019). Spatiotemporal railway use by grizzly bears in Canada's Rocky Mountains. J. Wildl. Manag..

[57] Van Why KR, Chamberlain MJ (2003). Mortality of black bears, *Ursus americanus*, associated with elevated train trestles. Can. Field-Nat..

[58] Cote SD, Rooney TP, Tremblay JP, Dussault C, Waller DM (2004). Ecological impacts of deer overabundance. Annu. Rev. Ecol. Evol. Syst..

[59] Sawyer H (2007). Habitat selection of Rocky Mountain elk in a nonforested environment. J. Wildl. Manag..

[60] Nielsen SE, Stenhouse GB, Boyce MS (2006). A habitat-based framework for grizzly bear conservation in Alberta. Biol. Conserv..

[61] Lamb CT, Mowat G, McLellan BN, Nielsen SE, Boutin S (2017). Forbidden fruit: Human settlement and abundant fruit create an ecological trap for an apex omnivore. J. Anim. Ecol..

[62] Fahrig L, Rytwinski T (2009). Effects of roads on animal abundance: An empirical review and synthesis. Ecol. Soc..

[63] Gundersen H, Andreassen HP (1998). The risk of moose *Alces alces* collision: A predictive logistic model for moose-train accidents. Wildl. Biol..

[64] McDonald LR, Messmer TA, Guttery MR (2019). Temporal variation of moose-vehicle collisions in Alaska. Hum.-Wildl. Interact..

[65] Schwartz CC, Franzmann AW (1992). Dispersl and survival of subadult black bears from the Kenai Penninsula, Alaska. J. Wildl. Manag..

[66] Pollock SZ, Nielsen SE, St Clair CC (2017). A railway increases the abundance and accelerates the phenology of bear-attracting plants in a forested, mountain park. Ecosphere.

[67] Steiner W, Leisch F, Hacklander K (2014). A review on the temporal pattern of deer-vehicle accidents: Impact of seasonal, diurnal and lunar effects in cervids. Accid. Anal. Prevent..

[68] Gaynor KM, Hojnowski CE, Carter NH, Brashares JS (2018). The influence of human disturbance on wildlife nocturnality. Science.

[69] Beaudry F, Demaynadier PG, Hunter ML (2010). Identifying hot moments in road-mortality risk for freshwater turtles. J. Wildl. Manag..

[70] Eberhardt E, Mitchell S, Fahrig L (2013). Road kill hotspots do not effectively indicate mitigation locations when past road kill has depressed populations. J. Wildl. Manag..

[71] Neumann W (2012). Difference in spatiotemporal patterns of wildlife road-crossings and wildlife-vehicle collisions. Biol. Conserv..

[72] Visintin C, van der Ree R, McCarthy MA (2016). A simple framework for a complex problem? Predicting wildlife-vehicle collisions. Ecol. Evolut..

[73] Falcucci A, Ciucci P, Maiorano L, Gentile L, Boitani L (2009). Assessing habitat quality for conservation using an integrated occurrence-mortality model. J. Appl. Ecol..

[74] Dorsey, B. P., Clevenger, A. & Rew, L. J. Relative risk and variables associated with bear and ungulate mortalities along a railroad in the Canadian Rocky Mountains. in *Railway Ecology* (eds L. Borda-de-Água, R. Barrientos, P. Beja, & H. M. Pereira) 135–155 (Springer, 2017).

[75] Jacoby ME (1999). Trophic relations of brown and black bears in several western North American ecosystems. J. Wildl. Manag..

[76] Andreassen HP, Gundersen H, Storaas T (2005). The effect of scent-marking, forest clearing, and supplemental feeding on moose-train collisions. J. Wildl. Manag..

[77] Babinska-Werka J, Krauze-Gryz D, Wasilewski M, Jasinska K (2015). Effectiveness of an acoustic wildlife warning device using natural calls to reduce the risk of train collisions with animals. Transp. Res. Part D-Transp. Environ..

[78] Seiler, A. & Olsson, M. Wildlife deterrent methods for railways—An experimental study. in *Railway Ecology* (eds L. Borda-de-Água, R. Barrientos, P. Beja, & H. M. Pereira) 277–291. 10.1007/978-3-319-57496-7 (Springer, 2017).

[79] Borda-de-Água, L., Barrientos, R., Beja, P. & Pereira, H. M. (eds) Railway ecology. in *Railway Ecology* 3–9. 10.1007/978-3-319-57496-7 (Springer, 2017).

[80] Barrientos R, Ascensao F, Beja P, Pereira HM, Borda-de-Agua L (2019). Railway ecology vs. road ecology: Similarities and differences. Eur. J. Wildl. Res..

